# Quantifying the influence of temperature on hand, foot and mouth disease incidence in Wuhan, Central China

**DOI:** 10.1038/s41598-018-20318-z

**Published:** 2018-01-31

**Authors:** Jiao Huang, Shi Chen, Yang Wu, Yeqing Tong, Lei Wang, Min Zhu, Shuhua Hu, Xuhua Guan, Sheng Wei

**Affiliations:** 1 Department of Epidemiology and Biostatistics, Ministry of Education Key Laboratory of Environment and Health, School of Public Health, Tongji Medical college, Huazhong University of Science and Technology, Wuhan, China; 2 Hubei Provincial Center for Disease Control and Prevention, Wuhan, China; 3 Department of Prevention and Health, Tongji Hospital, Tongji Medical College, Huazhong University of Science and Technology, Wuhan, China

## Abstract

Hand, foot and mouth disease (HFMD) is a substantial burden throughout Asia, but the effects of temperature pattern on HFMD risk are inconsistent. To quantify the effect of temperature on HFMD incidence, Wuhan was chosen as the study site because of its high temperature variability and high HFMD incidence. Daily series of HFMD counts and meteorological variables during 2010–2015 were obtained. Distributed lag non-linear models were applied to characterize the temperature-HFMD relationship and to assess its variability across different ages, genders, and types of child care. Totally, 80,219 patients of 0–5 years experienced HFMD in 2010–2015 in Wuhan. The cumulative relative risk of HFMD increased linearly with temperature over 7 days (lag0–7), while it presented as an approximately inverted V-shape over 14 days (lag0–14). The cumulative relative risk at lag0–14 peaked at 26.4 °C with value of 2.78 (95%CI: 2.08–3.72) compared with the 5^th^ percentile temperature (1.7 °C). Subgroup analyses revealed that children attended daycare were more vulnerable to temperature variation than those cared for at home. This study suggests that public health actions should take into consideration local weather conditions and demographic characteristics.

## Introduction

Hand, foot and mouth disease (HFMD) is an emerging infectious disease that is caused by several non-polio enteroviruses^1^. In China, HFMD is the most frequent infectious disease affected children under 5 years old^2^. Most HFMD cases have self-limiting symptoms, including fever, oral ulcers and maculopapular rashes or vesicular sores on the hands, feet, or buttocks^3^. However, a small portion of HFMD patients can develop serious health consequences, even death, due to complications from aseptic meningitis, encephalitis, acute flaccid paralysis, and neurogenic pulmonary edema^4^. Many studies on the epidemiology of HFMD have observed seasonality of HFMD incidence^5,6^. Furthermore, meteorological factors, particularly daily average temperature, have been reported to affect on the incidence of HFMD in recent studies.

Temperature as a key meteorological factor has been reported to be associated with HFMD occurrence in many countries. However, findings are inconsistent among studies. Several studies have demonstrated that the incidence of HFMD increased with rising temperature^7,8^, but a negative correlation has also been documented^9^. Several studies have reported non-linear effect of temperature on HFMD incidence and have found a cut-off point at 25.0–27.5 °C for the association between temperature and the risk of HFMD incidence^10,11^. In fact, different patterns of HFMD incidence have been found in studied areas with different climate conditions. For example, there is only one annual peak in summer in North China, while there are two peaks, in early summer and autumn, in South China. Since  temperature has a similar effect on enterovirus-associated HFMD, one question then arises: why are there different relationships between temperature and incidence of HFMD even within similar populations. The possible effect of temperature on HFMD incidence may be determined by a study in an area with high temperature variability and high incidence of HFMD. Wuhan is an ideal area for such study.

Wuhan is the capital of Hubei Province and the largest city in Central China with a population of over ten million. It is also the site of serious HFMD outbreaks and has over ten thousand HFMD cases reported each year. In addition, the weather in Wuhan is characterized by a hot and wet summer and a cold and humid winter. The summer in Wuhan is similar to that in South China, while the winter is similar to that in North China. The uniqueness of Wuhan’s climate is particularly suitable for studying the association between temperature and HFMD incidence. The aim of the present study was to quantify the effect of temperature on HFMD occurrence in Wuhan, and to assess whether this association varies by age, gender, and type of child care (children cared for at home, children attended daycare). A time-series analysis using surveillance data in Wuhan, China, was performed to identify the association of daily HFMD counts with temperature, which will assist in public health prevention and control measures.

## Results

### Data description

A total of 80,219 HFMD cases among patients aged 0–5 years were reported in Wuhan from 1 January 2010 to 31 December 2015. Only 7,280 cases (9.07%) were lab-confirmed. In this period, HFMD occurred more commonly in males with a male-to-female ratio of 1.67:1. The proportions of each age group were 14.4% (11,586 cases), 47.5% (38,088 cases) and 38.1% (30,545 cases) in children under 1 year old, aged 1–2 years and aged 3–5 years, respectively. There were 46,117 children (57.5%) cared for at home and 34,102 children (42.5%) who attended daycare. A descriptive summary of daily HFMD cases and meteorological variables is provided in Table 1. The mean values of average temperature and humidity were 16.7 °C and 78.7%, respectively. The time-series distribution of daily HFMD cases and meteorological variables in Wuhan from 2010 to 2015 revealed seasonality in HFMD occurrence (Supplementary Fig. S2). Summer peaks in the number of HFMD cases were observed in April-July, with secondary peaks occurring in November-December.

**Table 1:** Distribution of daily data on HFMD cases and meteorological variables in Wuhan, 2010–2015.

	Mean ± SD	Minimum	Q1	Median	Q3	Maximum
**NO. of HFMD cases**						
The entire cases	36.6 ± 33.4	0.0	12.0	24.0	50.5	195.0
Male	22.9 ± 20.9	0.0	8.0	15.0	32.0	122.0
Female	13.7 ± 13.0	0.0	4.0	9.0	19.0	89.0
<1 year old	5.3 ± 6.0	0.0	1.0	3.0	7.0	50.0
1–2 years old	17.4 ± 14.9	0.0	6.0	12.0	25.0	82.0
3–5 years old	13.9 ± 15.6	0.0	3.0	8.0	18.0	107.0
Children cared for at home	21.1 ± 18.4	0.0	7.0	14.0	30.0	125.0
Children attended daycare	15.6 ± 17.4	0.0	4.0	9.0	21.0	120.0
**Meteorological variables**						
Average temperature (°C)	16.7 ± 9.3	−2.9	8.6	17.8	24.7	33.2
Average press (hpa)	1012.8 ± 9.7	994.5	1004.8	1013.1	1020.0	1036.9
Average vaporpress (hpa)	16.9 ± 9.2	1.8	8.3	15.6	25.2	37.4
Average wind speed (m/s)	1.9 ± 1.0	0.0	1.1	1.7	2.4	7.2
Average humidity (%)	78.7 ± 10.9	28.0	73.0	80.0	86.0	100.0

### Relationship between daily average temperature and HFMD occurrence at different lags

**Figure 1:**
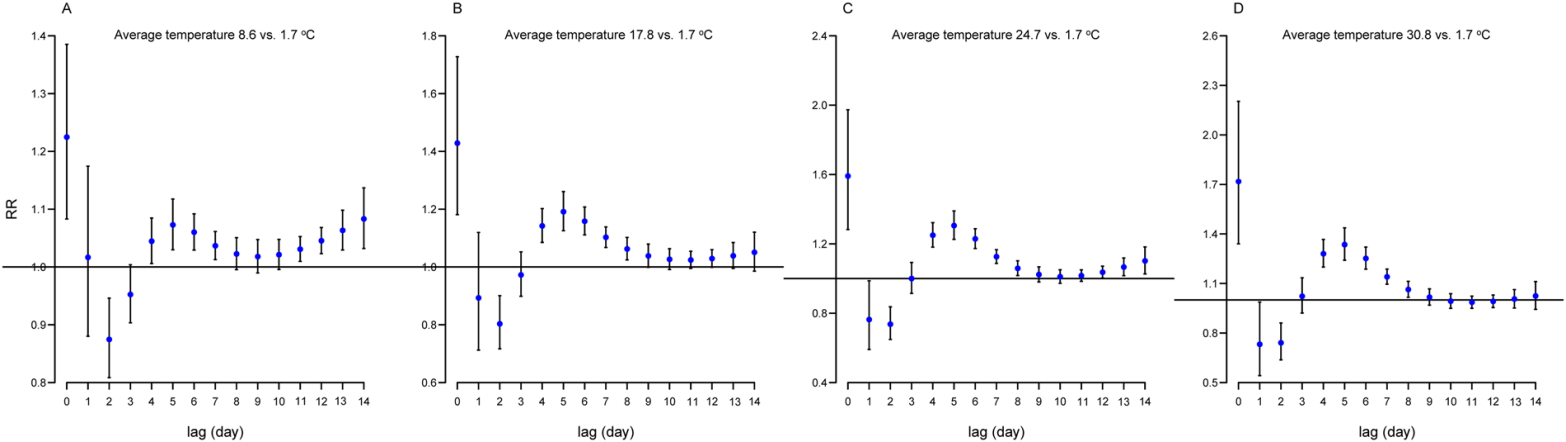
The relative risks of different average temperature for HFMD cases at different lags. The 5th percentile values of average temperature (1.7 °C) were defined as the reference for calculating relative risk. The temperature 8.6 °C, 17.8 °C, 24.7 °C and 30.8 °C represented the 25^th^ percentile, 50^th^ percentile, 75^th^ percentile and 95^th^ percentile of average temperature, respectively.

Figure 1 shows the effect of average temperature at different percentiles (25^th^, 50^th^, 75^th^ and 95^th^) on HFMD counts, compared with its 5^th^ percentile, at different lag days. The average temperature of the four above mentioned percentiles demonstrated a similar pattern of association with HFMD incidence. An immediate increase in relative risk (RR) at lag0 (the current day) was followed by a decrease at lag1 (the previous day) and lag2 (the previous two day). Subsequently, the RRs exhibited an inverted V-shape at lag2-lag14, with a peak at lag5. The RR values at lag5 of different percentiles (25^th^, 50^th^, 75^th^ and 95^th^) of temperature on HFMD incidence were 1.07 (95%CI: 1.03–1.12), 1.19 (95%CI: 1.13–1.26), 1.31 (95%CI: 1.23–1.39), and 1.34 (95%CI: 1.24–1.45), respectively.

### Cumulative RRs of average temperature on HFMD occurrence stratified by age, gender and type of child care

**Figure 2:**
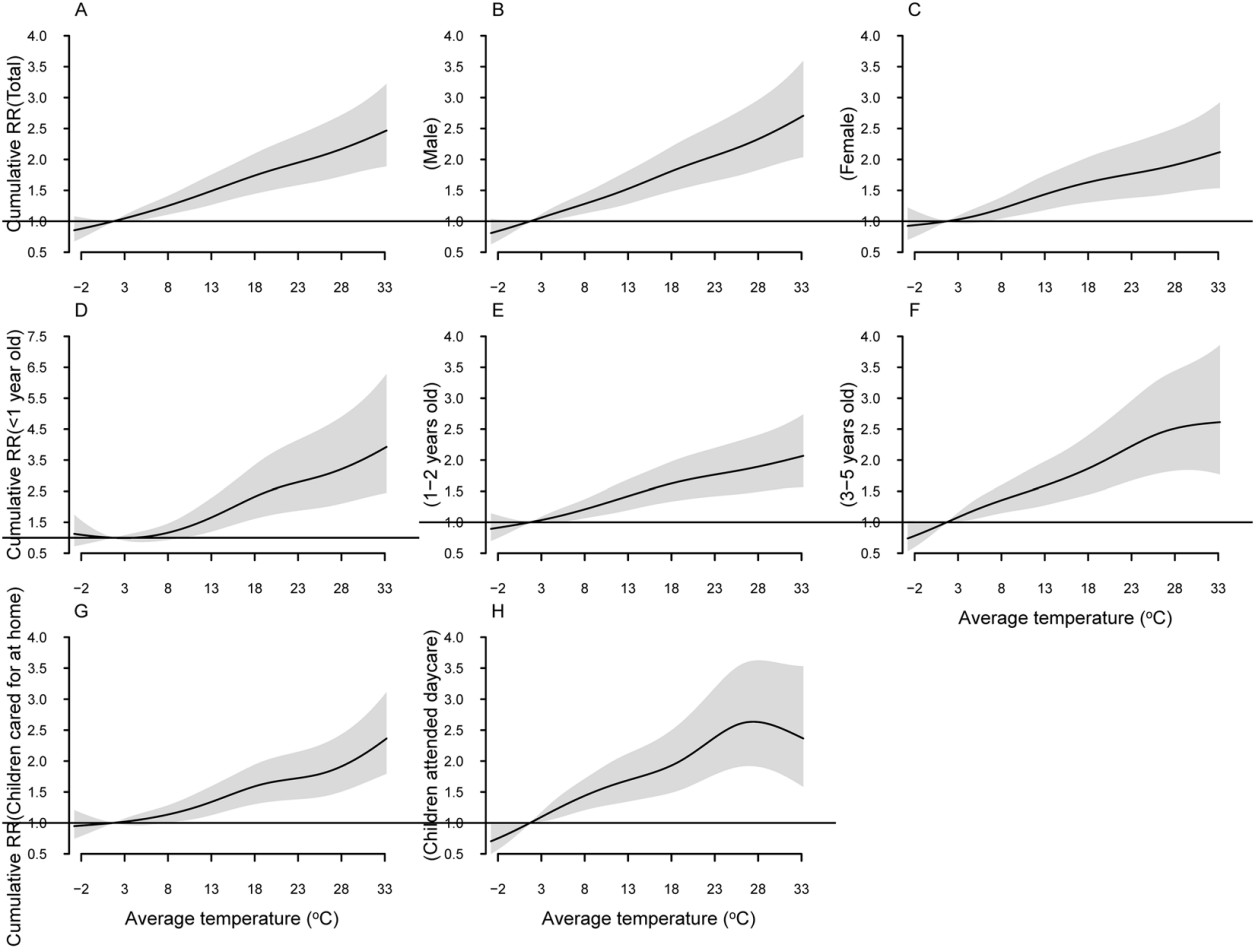
The cumulative relative risks of average temperature for the entire, gender-specific, age-specific and type of child care-specific HFMD cases over 7 days.

Figure 2 illustrates the cumulative RRs, aggregating all contributions up to 7 days of average temperature on HFMD incidence in different subgroups. The exposure-response relationship was shown to be linear in all subgroups, except for children who attended daycare. A positive association between average temperature and HFMD incidence was observed, with the cumulative RR for 1 °C rise being 4.51% (95CI: 3.12–6.30%) at lag0–7. A 1 °C increase in temperature elevated the risk of HFMD incidence by 5.16% (95%CI: 3.55–7.27%) and 3.49% (95%CI: 2.10–5.38%) in male and female subgroups. A 1 °C increase in temperature led to 8.74% (95%CI: 4.95–14.73%), 3.42% (95%CI: 2.21–5.03%), and 5.56% (95%CI: 3.39–8.67%) increases in HFMD incidence among children aged <1 year, 1-2 years, and 3–5 years, respectively. Each 1 °C increase in temperature was associated an increase of 3.74% (95%CI: 2.44–5.44%) in HFMD incidence among children cared for at home. For children who attended daycare, the cumulative RR increased with average temperature up to 27.5 °C and then began to decrease.

**Figure 3:**
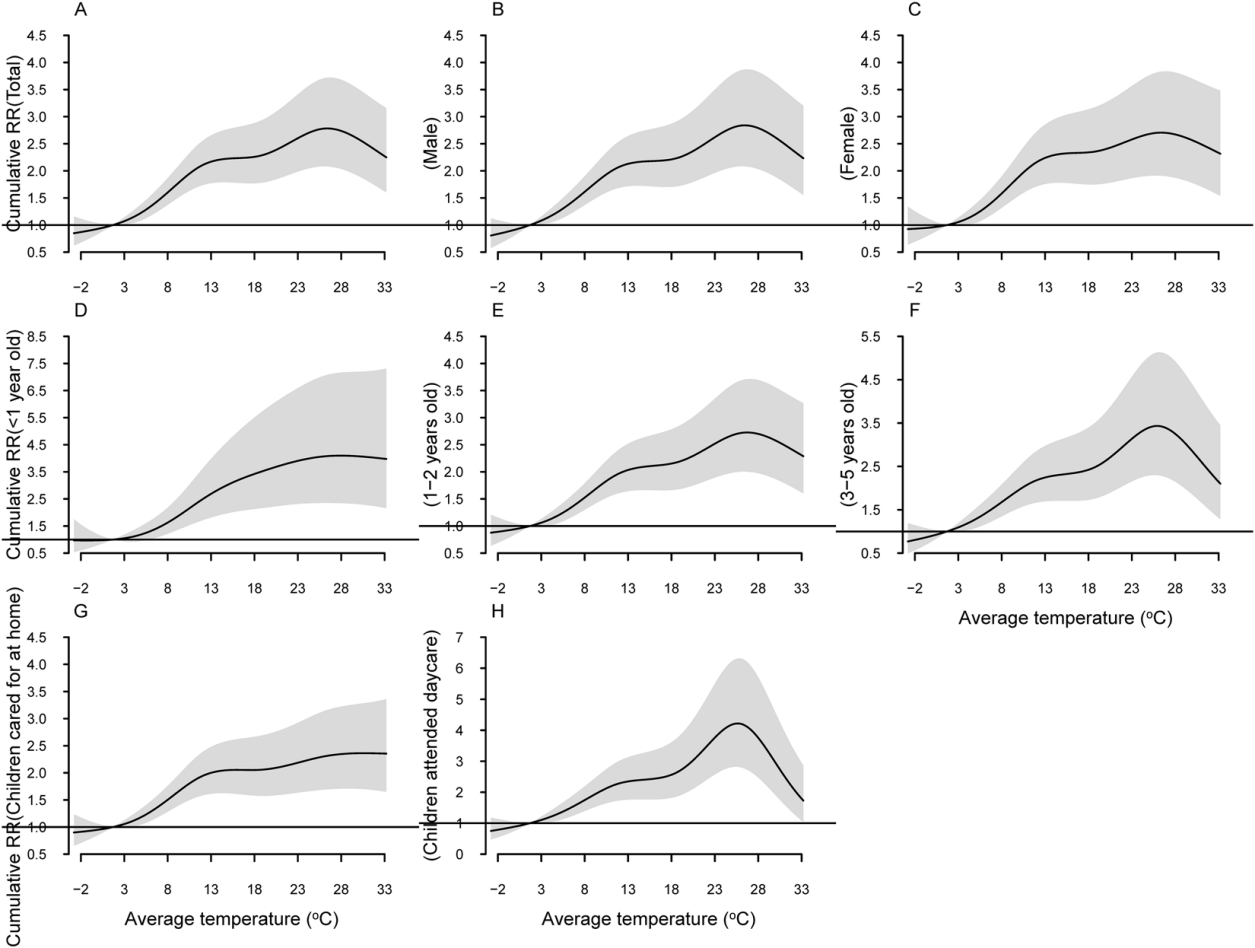
The cumulative relative risks of average temperature for the entire, gender-specific, age-specific and type of child care-specific HFMD cases over 14 days.

Figure 3 describes the cumulative RRs over 14 days of average temperature on HFMD incidence in different subgroups. The exposure-response relationship presented an approximately inverted V-shape. The cumulative RR reached a peak at 26.4 °C with a value of 2.78 (95%CI: 2.08–3.72). The overall effect of average temperature on HFMD occurrence in both males and females showed similar trends with respect to the entire study population. And it also presented to be an inverted V-shape, with different peak values for children in different age groups, i.e., 4.10 (95%CI: 2.35–7.16) at 27.9 °C for children younger than 12 months; 2.73 (95%CI: 2.00–3.71) at 26.7 °C for children aged 1-2 years old and 3.43 (95%CI: 2.30–5.13) at 25.9 °C for children aged 3-5 years old. The association between cumulative exposure to average temperature with HFMD incidence was approximately linear in children cared for at home, while it presented an inverted V-shape in children who attended daycare, with a peak cumulative RR of 4.22 (95%CI: 2.82–6.31) at 25.6 °C.

The cumulative RRs of different average temperatures on HFMD incidence along the lags in different subgroups are summarized in Table 2. Average temperature had higher cumulative RR on HFMD occurrence in children who attended daycare than that in children cared for at home (both *P* < 0.001 at lag0-7 and lag0–14). The differences of cumulative RRs of temperature on HFMD incidence between male and female were not significant (*P* = 0.977 at lag0–7 and *P* = 0.978 at lag0–14). Insignificant differences of cumulative RRs of temperature on HFMD incidence were observed in different age groups (*P* = 0.150 at lag0–7 and *P* = 0.289 at lag0–14).

**Table 2:** The cumulative relative risk of different average temperature for HFMD cases in different subgroups.

	Temperature				P*
	8.6 °C	17.8 °C	24.7 °C	30.8 °C	
**Lag0–7**					
The entire cases	1.28 (1.13, 1.45)	1.73 (1.44, 2.08)	2.02 (1.63, 2.50)	2.32 (1.83, 2.95)	
Male	1.31 (1.14, 1.50)	1.80 (1.48, 2.19)	2.14 (1.71, 2.69)	2.52 (1.95, 3.25)	0.977
Female	1.23 (1.06, 1.43)	1.62 (1.30, 2.02)	1.81 (1.40, 2.34)	2.02 (1.51, 2.69)	
<1 year old	1.21 (0.95, 1.53)	2.30 (1.60, 3.30)	2.91 (1.94, 4.38)	3.56 (2.30, 5.52)	0.150
1–2 years old	1.23 (1.08, 1.41)	1.62 (1.33, 1.96)	1.81 (1.44, 2.26)	1.98 (1.54, 2.55)	
3–5 years old	1.38 (1.16, 1.64)	1.85 (1.44, 2.38)	2.34 (1.74, 3.16)	2.58 (1.83, 3.63)	
Children cared for at home	1.15 (1.01, 1.31)	1.58 (1.30, 1.93)	1.76 (1.40, 2.22)	2.13 (1.66, 2.74)	<0.001
Children attended daycare	1.47 (1.23, 1.77)	1.92 (1.48, 2.48)	2.53 (1.87, 3.42)	2.52 (1.78, 3.57)	
**Lag0–14**					
The entire cases	1.69 (1.44, 1.97)	2.25 (1.77, 2.87)	2.73 (2.06, 3.62)	2.51 (1.84, 3.42)	
Male	1.69 (1.43, 2.00)	2.21 (1.70, 2.86)	2.77 (2.06, 3.74)	2.52 (1.81, 3.51)	0.978
Female	1.67 (1.38, 2.02)	2.34 (1.75, 3.13)	2.67 (1.90, 3.74)	2.50 (1.72, 3.64)	
<1 year old	1.74 (1.29, 2.36)	3.40 (2.12, 5.45)	4.02 (2.34, 6.88)	4.05 (2.28, 7.21)	0.289
1–2 years old	1.59 (1.36, 1.89)	2.14 (1.66, 2.77)	2.67 (1.97, 3.57)	2.51 (1.81, 3.49)	
3–5 years old	1.76 (1.41, 2.20)	2.41 (1.72, 3.37)	3.37 (2.28, 4.98)	2.66 (1.71, 4.13)	
Children cared for at home	1.58 (1.34, 1.85)	2.05 (1.58, 2.66)	2.26 (1.67, 3.05)	2.36 (1.70, 3.29)	<0.001
Children attended daycare	1.84 (1.46, 2.32)	2.54 (1.81, 3.57)	4.15 (2.80, 6.16)	2.61 (1.67, 4.08)	

(The 5^th^ percentile values of average temperature were defined as the reference for calculating relative risk.)

8.6 °C, 17.8 °C, 24.7 °C and 30.8 °C represented the 25^th^ percentile, 50^th^ percentile, 75^th^ percentile and 95^th^ percentile of average temperature, respectively.

*Multivariate Wald test.

### Sensitivity analyses

In the sensitivity analyses, when changing df (3–6) for temperature and humidity, we found that the patterns of temperature’s effect on HFMD incidence at different lags (Supplementary Fig. S3), cumulative RRs over 7 days and 14 days did not vary substantially (Supplementary Fig. S4 and Supplementary Fig. S5, respectively). When the maximum lag was set to different values (13–21 days), the effect of temperature on HFMD incidence presented similar patterns (Supplementary Figs. S6–S8).

## Discussion

This study provides evidence of association between daily average temperature and HFMD occurrence among children aged 0–5 years using population-wide monitoring data in Wuhan, China. After adjusting other climate variables, the relationship was found to be linear between cumulative exposure to average temperature over 7 days and HFMD occurrence, while it appeared to be an inverted V-shape when cumulative exposure to average temperature amounted to 14 days, with a peak in cumulative RR at 26.4 °C observed for the entire study population. Children who attended daycare were more sensitive to temperature variation than those cared for at home. The cumulative relative risks of temperature on HFMD incidence were not found to be significantly different in different age and gender groups. The results of this study may be informative in forecasting HFMD risk for children with different types of child care based on daily average temperature change.

Temperature influences the survival and spread of infectious pathogens in the environment, as well as the behavior and activities of the population, thereby influencing the dynamics of infection transmission^12,13^. Several laboratory-based studies have shown that the stability of enteric viruses is influenced by temperature and relative humidity^14,15^. Warm seasons may extend virus survival and improve virulence in the environment; such changes could increase the opportunities for hosts to become infected following contact with contaminated surfaces^16^. In addition, several studies have indicated that temperature is connected to changes in human contact behaviors, which could affect the incidence of HFMD^17,18^. For example, during warmer periods, people are more likely to spend their time outside rather than stay at home. This may contribute to the spread of enteroviruses through respiratory droplets, ruptured skin vesicles, or direct contact with contaminated toys and environmental surfaces, all of which increase the frequency of contact, and therefore the occurrence of HFMD.

The pattern of association between temperature and HFMD risk is not consistent among studies. Our study suggested a linear relationship over 7 days and an inverted V-shape over 14 days. The association between temperature and HFMD occurrence has been reported to be linear in several regions, such as Guangzhou^19^, Rizhao^20^, Hong Kong^21^, Japan^22^ and Shenzhen^7^. The excessive relative risk of HFMD was 2.75% for 1 °C increase in average temperature at lag6 in Shenzhen^7^. But the present study found 4.51% increase in HFMD incidence for 1 °C rise in temperature at lag0–7 in Wuhan. The larger estimates of HFMD risk in Wuhan may partly due to cumulative effect for the previous 7 days rather than a single day. Nevertheless, non-linear relationships similar to the relationship indicated in the present study have also been documented in several studies^23–25^. Different average temperature ranges may be the reason for this inconsistency. The mean values of average temperature varied from 13 °C to 23 °C in these study regions. One study reported an increased association between average temperature and HFMD incidence below 26.2 °C, but a decreased association above 26.2 °C in Beijing^23^. The incidence of HFMD rose at temperatures above 14 °C; at temperatures above approximately 23 °C, it began to decline, producing an inverted V-shaped relationship in Chengdu^25^. The thresholds of average temperature differed between these studies, potentially due to the different serotypes of enterovirus circulating during the study period. The discrepancy could also be attributed to different climatic and geographic conditions among the cities. Despite these differences, the same pattern of association between average temperature and the incidence of HFMD revealed that the appropriate average temperature for an HFMD outbreak in Wuhan is approximately 26 °C. Higher temperatures could lead to the activation of enteroviruses and affect children’s activities, reducing the time spent outdoors and thus the possibility of contact with other children^26^.

We observed that types of child care may be an important modifier of temperature effect on HFMD incidence. In spite of insignificant difference, stratified analyses indicated that children younger than 1 year old and those aged 3–5 years (children who attended daycare) were more vulnerable to temperature change on HFMD incidence than those aged 1–2 years. A recent study also indicated that children younger than 1 year old were more sensitive to temperature^25^. It has been reported that the positive rate of maternal EV-A71-neutralizing antibody was 48–85% at birth^27–29^ and decreased to 1–7% at 6 months^27,29,30^. After that, the positive rate of natural infection increased gradually with age, and stayed stable at 50 to 80% for children aged 6 years or older^31–33^. Therefore, the susceptibility of children aged 0–1 years to temperature may increase because of the lack of immunity. A study in Taiwan suggested that attending a daycare center or kindergarten significantly increased the likelihood of EV-A71 infection^34^. It is important to note that children’s activities and behaviors differ across different age groups. Personal hygiene practices among children aged 3–5 years are sub-optimal, and proper hygienic supervision of children in kindergarten is difficult for caregivers. Children of this age subgroup are generally cared for at preschool or kindergarten, where they may share beds during noontime breaks and toys, which can be easily contaminated with enteroviruses.

The present study provides a better understanding of the relationship between temperature variation and HFMD risk in Wuhan. Even though it could not fully capture HFMD dynamics from a causal perspective, the finding could serve as an effective way for identifying the risk of HFMD incidence based on short-term weather change. Thus, prevention measures such as good hygiene practices and temporary closure of educational institutions could be adopted before the actual upsurge of HFMD occurrence and thus prevent epidemics in a local scale. Moreover, this study found that children attended daycare (aged 3–5 years old) were more susceptible to temperature variation on HFMD incidence. This helps us enhance measures focused on this specific population and inform parents or caregivers to be aware of HFMD occurrence when temperature changed.

However, some limitations of this study should be acknowledged. First, our study was an ecological one, which did not permit us to explore the association based on individuals and inhibit assessment of causal inference. Second, the present study was focused in one city, and the results may not be applicable to other locations, particularly those with different climates. Third, air pollutants, such as PM_10_, may play an important role in HFMD risk^35^. Since we have not available air pollutants data for the present study, we have not discussed its effect on the HFMD risk. Further study is needed to address air pollutants’ roles on HFMD risk. Furthermore, this study did not analyze the pathogenic origin of HFMD as EV-A71, CV-A16 or other enteroviruses. It has been reported that in some regions, EV-A71 and CV-A16 are the predominant viruses causing HFMD in spring and summer, while other enteroviruses cause HFMD during autumn and winter^36^. Future research should therefore concentrate on the relationship between enterovirus serotypes and meteorological variables. Despite these limitations, our findings contribute to the understanding of temperature effects on HFMD incidence, which may inform health authorities in the development of efficient prevention strategies.

In conclusion, this study suggests that daily average temperature may be an important predictor of HFMD occurrence in different subgroups of children in Wuhan, China. It could be helpful in guiding health resource allocation and development of public health preparedness and intervention strategies.

## Materials and Methods

### Study area

Wuhan, the capital city of Hubei Province, is located in Central China and features a land size of 8,569.15 km2 and population density of 1,206 persons per km^2^ as of 2014. It experiences a subtropical monsoon climate, with an annual average temperature of 16.7 °C and relative humidity of 81%^37^.

### Data sources

HFMD has been listed among the notifiable infectious diseases in China since May 2008, and cases must be reported via the national enhanced surveillance system in mainland China. The system data are collected nationwide and are therefore more representative of an actual HFMD epidemic. A 2011 data-quality inspection report indicated that the collected data are of high quality, particularly in the eastern regions of China, with reporting completeness of 99.84% and accuracy of information reported of 92.76%^38^. In our study, daily reported cases of HFMD in Wuhan from January 2010 to December 2015 were obtained from the Hubei Provincial Center for Disease Control and Prevention. Patients were diagnosed with HFMD if they presented with the following symptoms: scattered vesicular lesions on oral mucosa, papules and vesicular lesions on palms, soles and, occasionally, the buttocks with or without fever in accordance with the National Guidelines on clinical management of Hand, Foot and Mouth Disease disseminated by the Chinese Ministry of Health^39^. If patient presented with laboratory evidence of enterovirus infection (including EV-A71, CV-A16, or other non-EV71 and non-CV-A16 enterovirus), he or she was defined as a lab-confirmed case. All HFMD cases were requested to be reported to the system online within 24  hours of diagnosis. The gender, age, and child care types of HFMD patients were also obtained. Because 94.9% of HFMD cases occurred in children aged 0–5 years in Wuhan, only children of this age group were chosen for the present study. Daily meteorological data, including average temperature, average air pressure, average vapor pressure, average relative humidity and average wind speed for the same period, were obtained from the China Meteorological Data Sharing Service System (http://www.cma.gov.cn/).

### Statistical analyses

Meteorological data and HFMD cases were linked by date and analyzed using a time-series design. A distributed lag non-linear model (DLNM) was constructed to represent a modeling framework to flexibly describe associations showing potentially non-linear and delayed effects in time-series data^40,41^. In the present study, DLNM combined with a quasi-Poisson distribution was performed to quantify the relationship between daily temperature and HFMD counts adjusted with other meteorological variables. The model was specified as follows:

$$\begin{array}{c}{\rm{logE}}({{\rm{Y}}}_{{\rm{t}}})={\rm{\alpha }}+\sum {\rm{cb}}[({\rm{meteorological}}\,\text{factors},\,{{\rm{df}}}_{1}),(\text{lag},\,{{\rm{df}}}_{2})]+{\rm{ns}}({\rm{DOY}},\,{\rm{df}}=6)\\ +\text{ns}(\text{time},\,{\rm{df}}=4)+{\rm{DOW}}+{\rm{PH}}+{\rm{autoregressive}}\,{\rm{term}}\end{array}$$

where E(Yt) was the expected number of HFMD cases on day t; α was the intercept; cb denoted the cross-basis function; df was the degree of freedom; ns() indicated a smooth function based on natural cubic splines; DOY referred to the day of the year, while time represented the day of the entire study period; DOW referred to the day of the week; PH presented a binary variable for public holidays. An autoregressive term for daily HFMD cases was also included in the model to account for autoregression. The relative risks (RRs) of a chosen percentile value of a meteorological variable with 95% confidence intervals (CI), compared with its 5^th^ percentile, were estimated. We also assessed the cumulative exposure-response relationship accounting for the entire lag period.

To investigate the effect of the meteorological variables on HFMD occurrence in different subgroups, stratified analyses were performed for subgroups of different ages (<1, 1–2, 3–5 years), types of child care (children cared for at home, children attended daycare) and genders (male and female). Significance test for subgroup analysis was based on a multivariate Wald test of the coefficients^42^. These sets of coefficients represented the change in the overall cumulative exposure–response curves in each potential effect modifier (such as male and female). The null hypothesis was that no difference for subgroups.

A preliminary analysis was performed to explore the correlation between meteorological variables by a Spearman’s rank correlation analysis. Air pressure and vapor pressure were not included in the model because of high correlation with temperature, while wind speed was excluded because of insignificant association with HFMD counts (Supplementary Table S1). Therefore, only temperature and humidity were incorporated into the models. The maximum lag was set to 14 days, given the incubation period of HFMD and our data’s suggestion that the relationships between temperature and childhood HFMD were negligible after a 14-day lag. The cumulative effect of temperature on HFMD over a lag of 0–7 days has also been estimated, as HFMD has an incubation period of 3–7 days. In our preliminary model, the degree of freedom in the smoothing function of covariables, i.e., temperature, humidity, DOY and time, and lag, was selected based on previous studies. Specifically, the cross-basis function was applied to temperature and humidity to model the non-linear exposure association with 5 df and the lagged association with 4 df^23,43^. DOY with 6 df was applied to control for seasonality, while time with 4 df was adjusted for long-term trends^10,44^. DOW and PH were incorporated into the models to control for the fluctuation in one week and public holidays. An autoregressive term for lag1–7 days of daily HFMD cases was included in the models based on partial autocorrelation function plot (Supplementary Fig. S1).

Because the effect estimation may vary with parameters specification in the model, the following sensitivity analyses were performed: (1) varying the df (3–6) for temperature and relative humidity; (2) varying the maximum lag (13–21 days). Quasi Akaike Information Criterion (QAIC) was used to assess the model fits and ascertain the final model parameters. All data manipulation and analyses were performed using the “dlnm”, “mgcv” and “spline” packages in the R statistical software (Version 3.2.5, http://cran.r-project.org).

### Ethical approval

HFMD has been listed among the notifiable infectious diseases in China since May 2008. According to the national surveillance protocol in China, the collection of individual data for all notifiable diseases, including HFMD, was part of an ongoing public health response and was thus exempt from institutional review board assessment. The information contained in the patients’ records was anonymized and deidentified prior to analysis. Only aggregated data were analyzed and reported.

### Data availability

The datasets generated during and/or analyzed during the current study are available from the corresponding author on reasonable request.

## Electronic supplementary material

Supplementary Information
