# Supplementary material for: Quantifying the influence of temperature on hand, foot and mouth disease incidence in Wuhan, Central China
Source: Sci Rep. 2018 Jan 31;8:1934. doi: 10.1038/s41598-018-20318-z (PMC5792432; doi:10.1038/s41598-018-20318-z)
Supplement: Supplementary file 1 — Supplementary Information [file 41598_2018_20318_MOESM1_ESM.pdf]

Quantifying the influence of temperature on hand, foot and mouth disease incidence in Wuhan, Central China

Jiao Huang[1], Shi Chen[1], Yang Wu[2], Yeqing Tong[2], Lei Wang[2], Min Zhu[1], Shuhua Hu[3], Xuhua Guan[2], Sheng Wei[1]*

Author affiliations:

[1]Department of Epidemiology and Biostatistics, Ministry of Education Key Laboratory of Environment and Health, School of Public Health, Tongji Medical college, Huazhong University of Science and Technology, Wuhan, China

[2]Hubei Provincial Center for Disease Control and Prevention, Wuhan, China.

[3]Department of Prevention and Health, Tongji Hospital, Tongji Medical College, Huazhong University of Science and Technology, Wuhan, China

***Corresponding author:** Sheng Wei

Sheng Wei, MD, PhD. Department of Epidemiology and Biostatistics, Ministry of Education Key Laboratory of Environment and Health, School of Public Health, Tongji Medical college, Huazhong University of Science and Technology, Wuhan, Hubei, China.

Tel: 86-27-83692031; Fax: 86-27-83692031;

E-mail: ws2008cn@gmail.com; ws1998@hotmail.com

Table S1. Spearman correlation coefficients between daily meteorological variables and HFMD cases, 2010-2015

	Average temperature	Average press	Average vapor press	Average wind speed	Average humidity	HFMD cases
Average temperature	1					
Average press	-0.90*	1				
Average vapor press	0.97*	-0.90*	1			
Average wind speed	0.08*	-0.10*	0.06*	1		
Average humidity	-0.03	-0.10*	0.19*	-0.13*	1	
HFMD cases	0.32*	-0.30*	0.31*	-0.04	0.05*	1

*p<0.05

Suppl Fig. S1

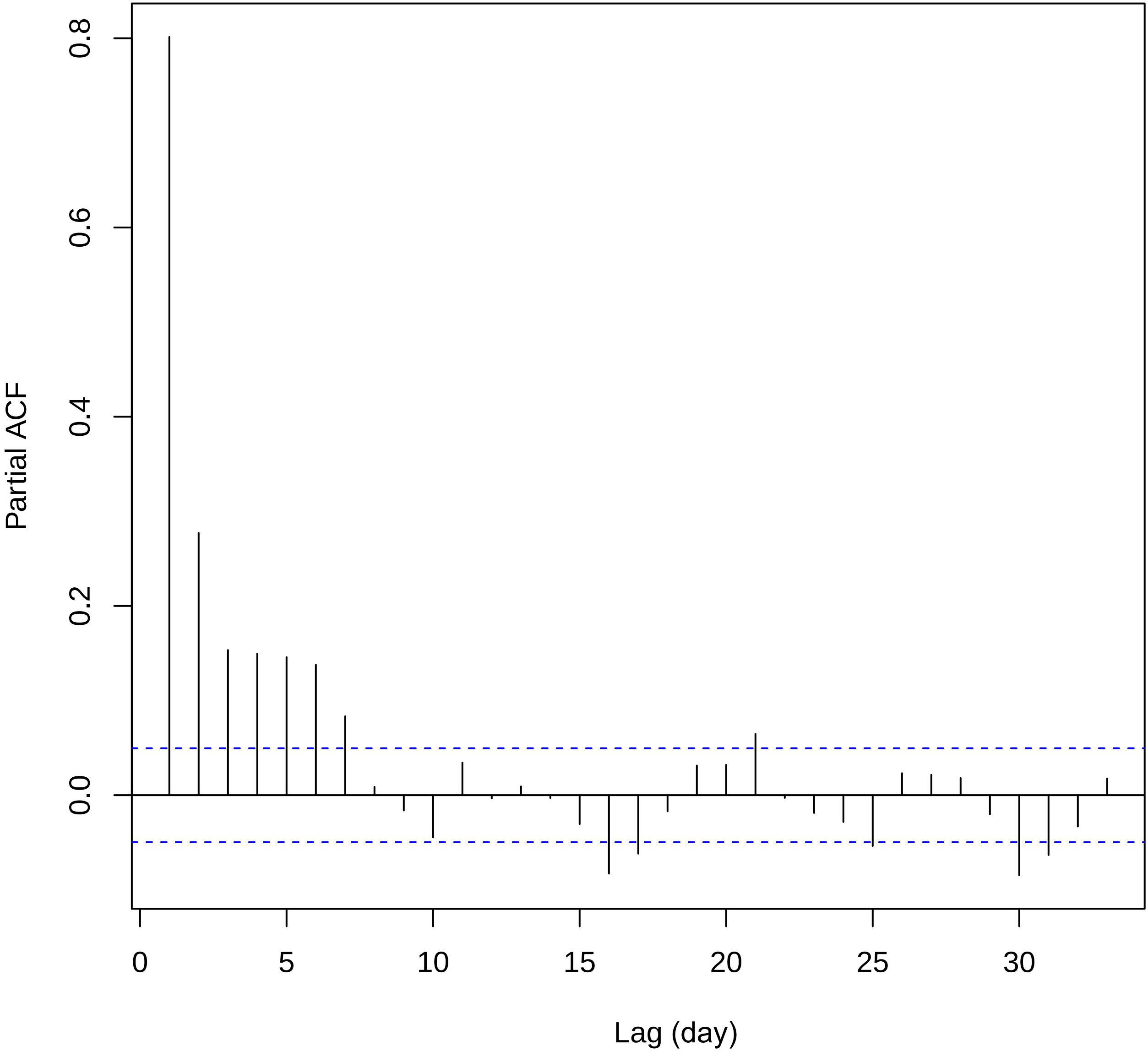

Suppl Fig. S2

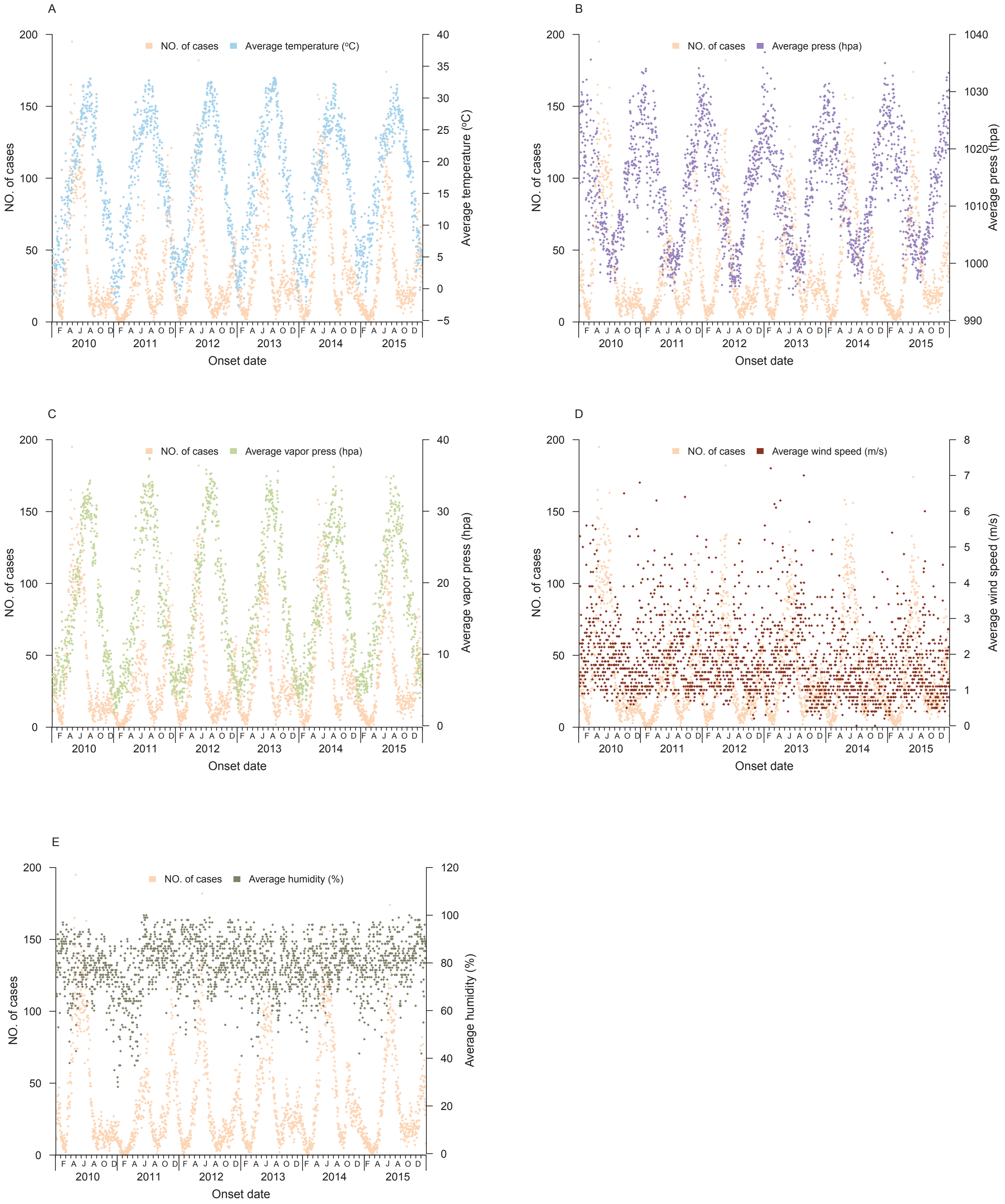
A
NO. of cases
Average temperature (ºC)
B
Average press (hpa)
C
Average vapor press (hpa)
D
Average wind speed (m/s)
E
Average humidity (%)
F A J A O D
2010
2011
2012
2013
2014
2015
Onset date

Suppl Fig. S3

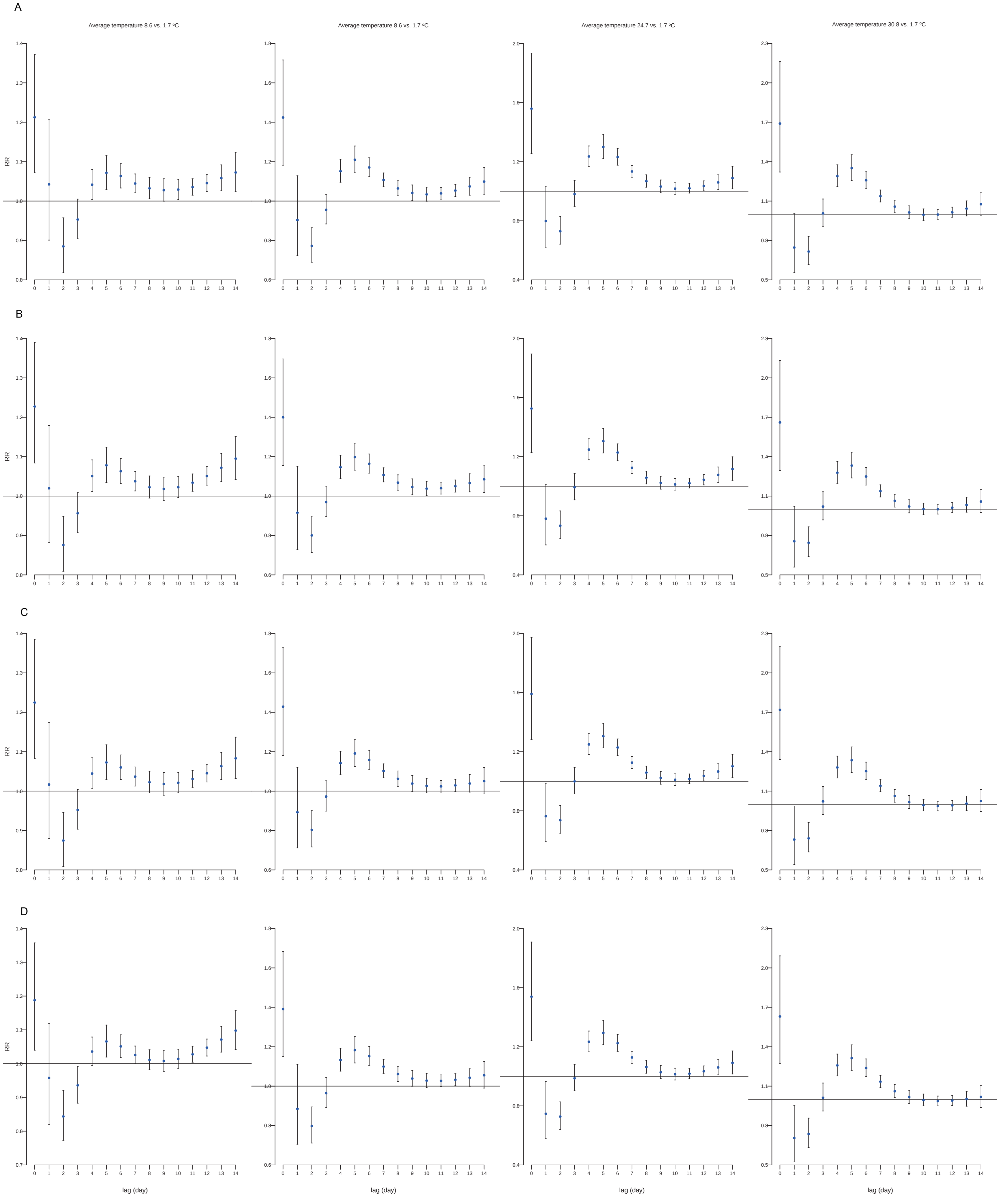

A
Average temperature 8.6 vs. 1.7 ºC
Average temperature 8.6 vs. 1.7 ºC
Average temperature 24.7 vs. 1.7 ºC
Average temperature 30.8 vs. 1.7 ºC
B
C
D
RR
lag (day)

Suppl Fig. S4

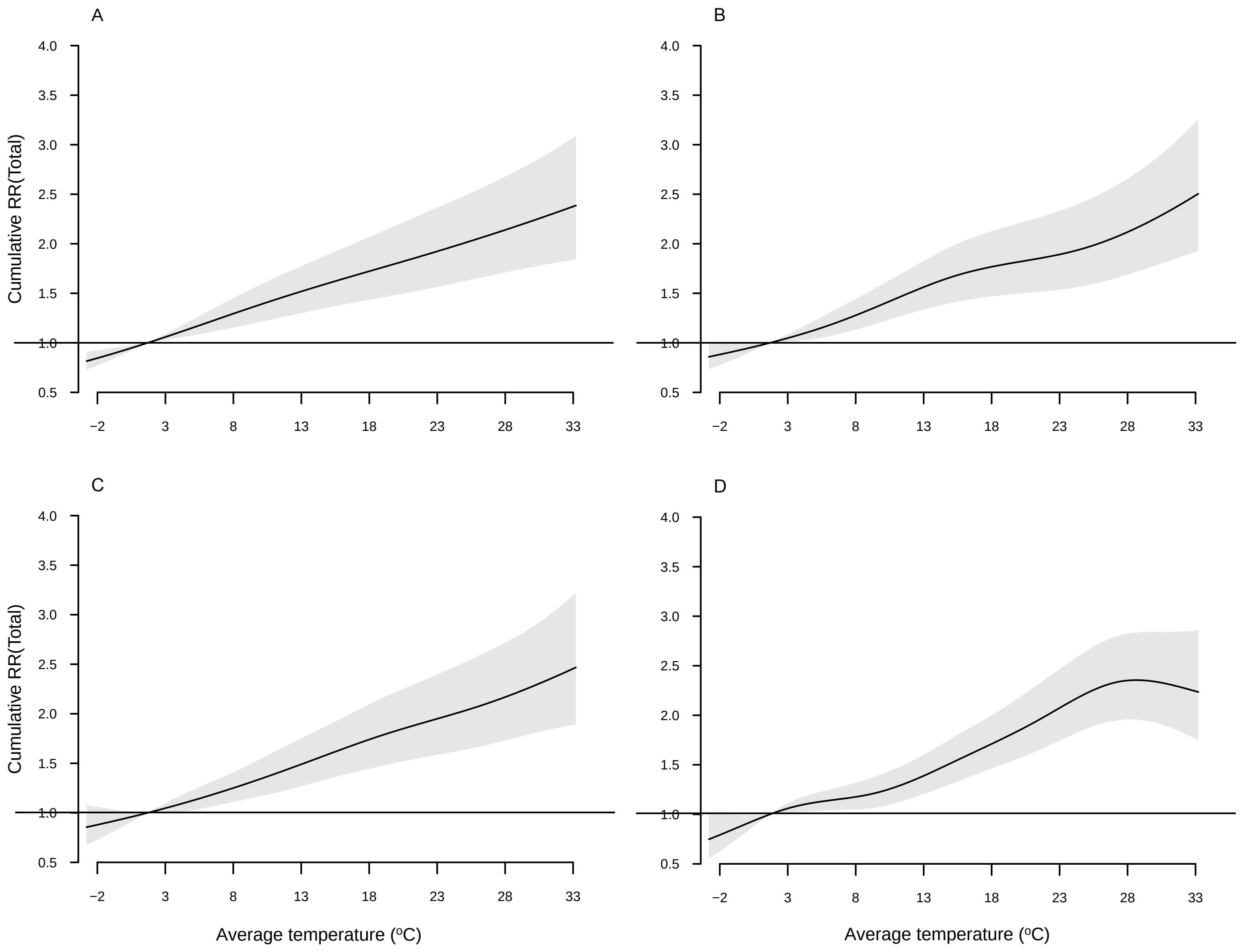
A
B
C
D
Cumulative RR(Total)
4.0
3.5
3.0
2.5
2.0
1.5
1.0
0.5
−2
3
8
13
18
23
28
33
Average temperature (ºC)

Suppl Fig. S5

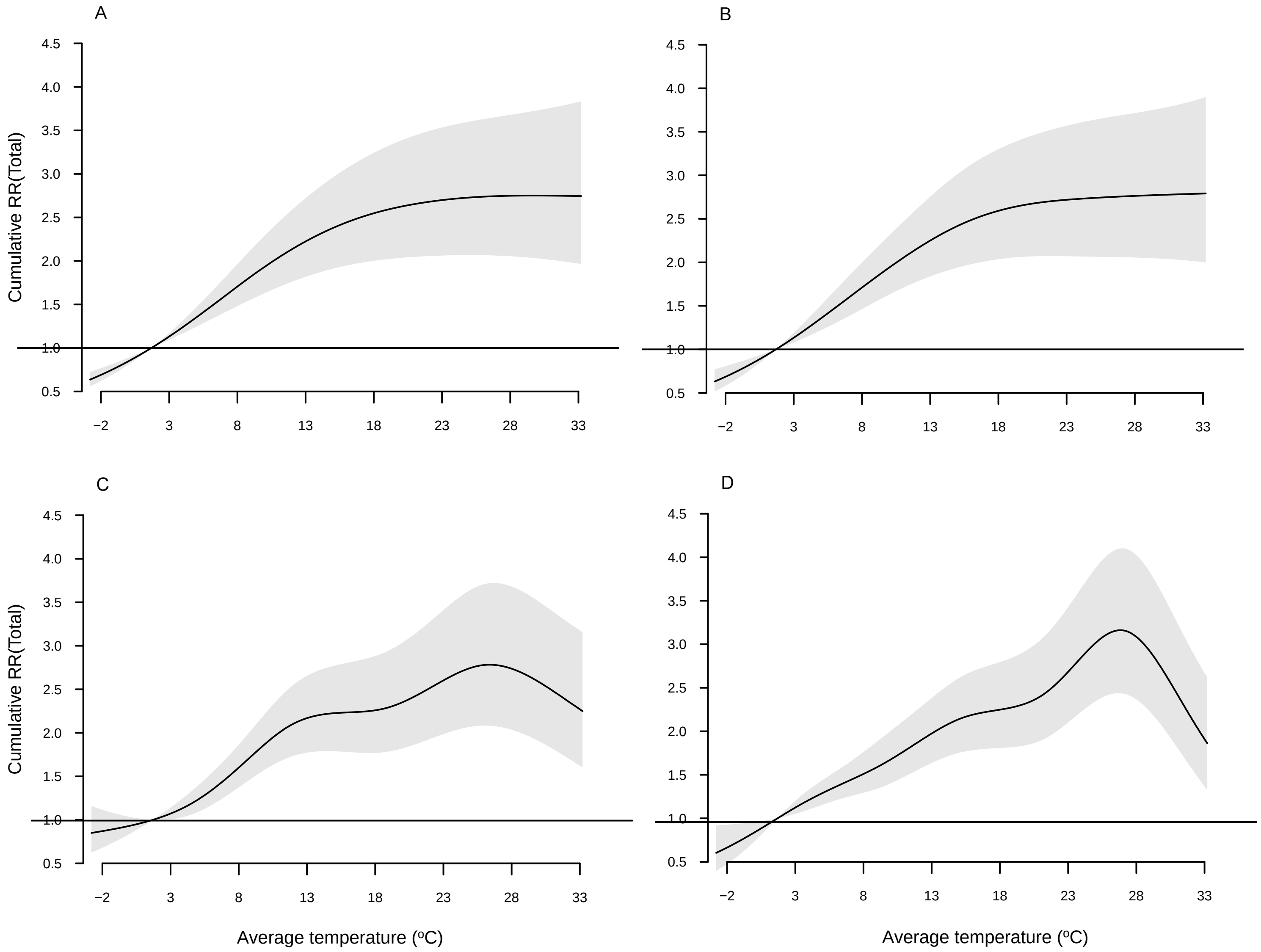
A
B
C
D
Cumulative RR(Total)
Cumulative RR(Total)
4.5
4.0
3.5
3.0
2.5
2.0
1.5
1.0
0.5
−2
3
8
13
18
23
28
33
Average temperature (ºC)
Average temperature (ºC)

Suppl Fig. S6

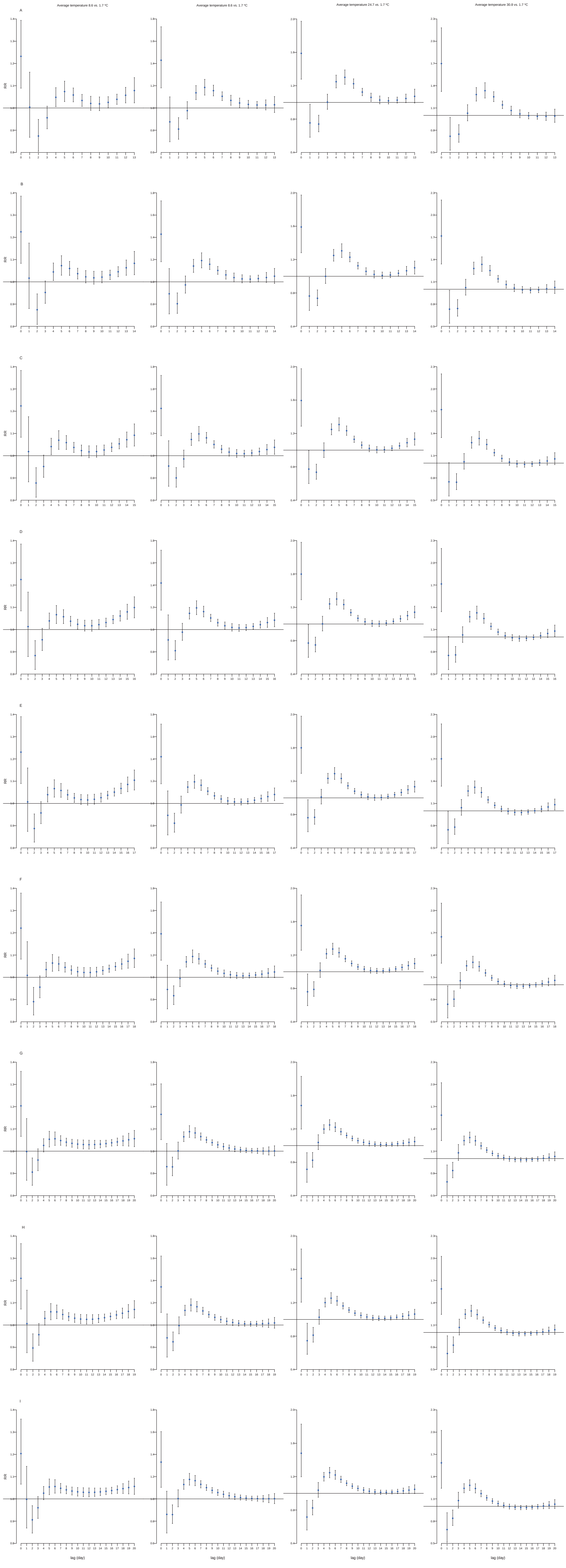
Average temperature 8.6 vs. 1.7 °C
Average temperature 8.6 vs. 1.7 °C
Average temperature 24.7 vs. 1.7 °C
Average temperature 30.8 vs. 1.7 °C
A
B
C
D
E
F
G
H
I
RR
lag (day)

Suppl Fig. S7

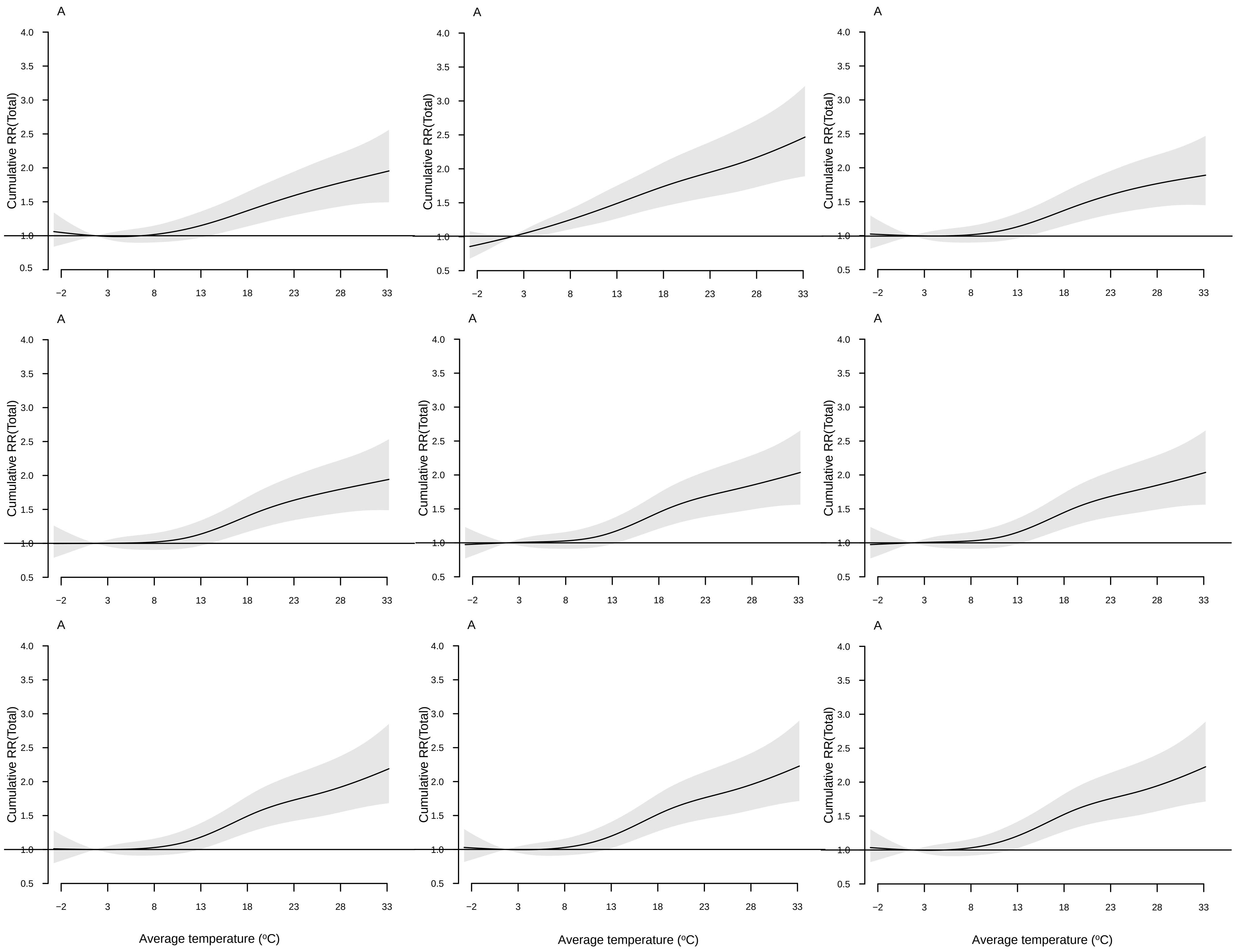
A
Cumulative RR(Total)
4.0
3.5
3.0
2.5
2.0
1.5
1.0
0.5
−2
3
8
13
18
23
28
33
Average temperature (ºC)

Suppl Fig. S8

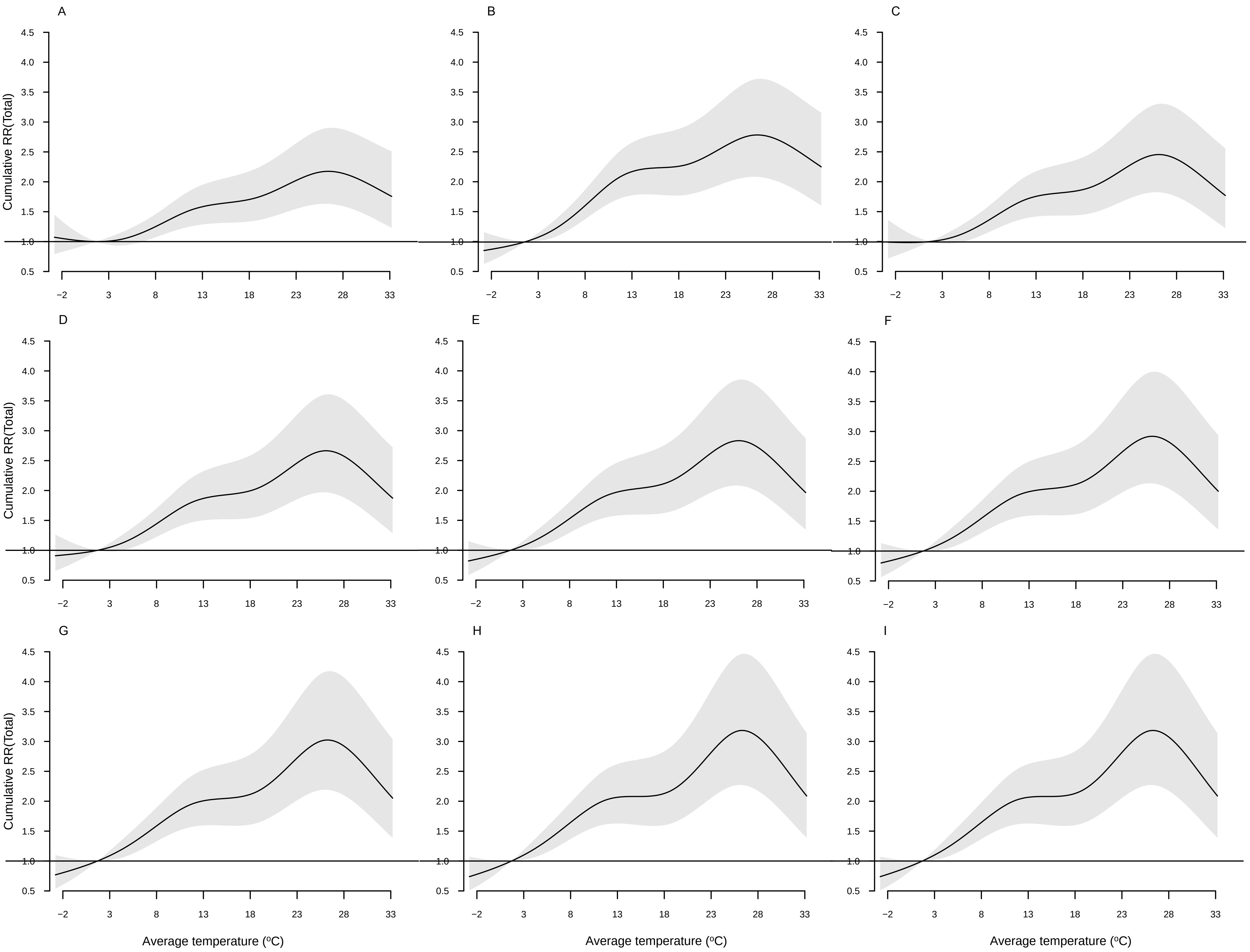
A
B
C
D
E
F
G
H
I
Cumulative RR(Total)
4.5
4.0
3.5
3.0
2.5
2.0
1.5
1.0
0.5
−2
3
8
13
18
23
28
33
Average temperature (ºC)

Supplementary Figure Legends

Supplementary Fig. S1 online: Partial auto-correlation function for residuals after seasonality and long-term trends have been removed (DOY with 6 df and time with 4 df).

Supplementary Fig. S2 online: Time series of daily HFMD cases and meteorological variables in Wuhan, 2010-2015.

Supplementary Fig. S3 online: The relative risks of different average temperature for HFMD cases at different lags, when changing the df (3-6) for temperature and relative humidity. Panel A: df=3, B: df=4, C: df=5, D: df=6

Supplementary Fig. S4 online: The cumulative relative risks of average temperature for the entire HFMD cases over 7 days, when changing the df (3-6) for temperature and relative humidity. Panel A: df=3, B: df=4, C: df=5, D: df=6

Supplementary Fig. S5 online: The cumulative relative risks of average temperature for the entire HFMD cases over 14 days, when changing the df (3-6) for temperature and relative humidity. Panel A: df=3, B: df=4, C: df=5, D: df=6

Supplementary Fig. S6 online: The relative risks of different average temperature for HFMD cases at different lags, when changing the maximum lag (13-21 days).

Panels A-I indicate maximum lags 13-21, respectively.

Supplementary Fig. S7 online: The cumulative relative risks of average temperature for the entire HFMD cases over 7 days, when changing the maximum lag (13-21 days). Panels A-I indicate maximum lags 13-21, respectively.

Supplementary Fig. S8 online: The cumulative relative risks of average temperature for the entire HFMD cases over the maximum lag days, when changing the maximum lag (13-21 days). Panels A-I indicate maximum lags 13-21, respectively.
